# PTPRK suppresses progression and chemo‐resistance of colon cancer cells via direct inhibition of pro‐oncogenic CD133

**DOI:** 10.1002/2211-5463.12636

**Published:** 2019-04-18

**Authors:** Masashi Matsushita, Yusuke Mori, Kyosuke Uchiumi, Takehiro Ogata, Mizuyo Nakamura, Hiroyuki Yoda, Hiroaki Soda, Nobuhiro Takiguchi, Yoshihiro Nabeya, Osamu Shimozato, Toshinori Ozaki

**Affiliations:** ^1^ Laboratory of DNA Damage Signaling Chiba Cancer Center Research Institute Japan; ^2^ Laboratory of Oncogenomics Chiba Cancer Center Research Institute Japan; ^3^ Laboratory of Cancer Genetics Chiba Cancer Center Research Institute Japan; ^4^ Department of Esophago‐Gastrointestinal Surgery Chiba Cancer Center Hospital Japan

**Keywords:** CD133, colon carcinoma, drug resistance, protein tyrosine phosphatase, PTPRK

## Abstract

Receptor‐type protein tyrosine phosphatase κ (PTPRK) is considered to be a candidate tumor suppressor. PTPRK dephosphorylates CD133, which is a stem cell marker; phosphorylated CD133 accelerates xenograft tumor growth of colon cancer cells through the activation of AKT, but the functional significance of this has remained elusive. In this study, we have demonstrated that knockdown of *PTPRK* potentiates the pro‐oncogenic CD133–AKT pathway in colon cancer cells. Intriguingly, depletion of *PTPRK* significantly reduced sensitivity to the anti‐cancer drug oxaliplatin and was accompanied by up‐regulation of phosphorylation of Bad, a downstream target of AKT. Together, our present observations strongly suggest that the CD133–PTPRK axis plays a pivotal role in the regulation of colon cancer progression as well as drug resistance.

**Abbreviations**
CSC: cancer stem cell
EGF: epidermal growth factor
EGFR: epidermal growth factor receptor
PARP: poly(ADP‐ribose) polymerase
PTP: protein tyrosine phosphatase
PTPRK: receptor‐type protein tyrosine phosphatase κ
ROS: reactive oxygen species
shRNA: short hairpin RNA

Colon cancer is the third leading cause of cancer‐related death and responsible for over 50 000 per year deaths in the USA 1. Although there has been substantial progress in treatment in the past decade, relapse and metastasis to the other tissues/organs are serious issues to be addressed to cure colon cancer patients 2. Various phase 3 clinical trials to evaluate the potential effects of doublet or triplet chemotherapy plus molecular targeting agents such as bevacizumab showed an improvement in the progression‐free survival period of patients with advanced colon cancer; however, their 5‐year survival rate has not been improved 3. In this regard, understanding the precise molecular mechanism(s) behind the aggressiveness and resistance to therapy of advanced colon cancer is an urgent issue to be adequately addressed for better treatment of colon malignancy.

A growing body of evidence suggests that a tyrosine phosphorylation of cellular proteins is one of the critical molecular events regulating numerous cellular processes such as proliferation and cell death; therefore, its abnormality might disrupt the strict regulation of these processes, thereby triggering the initiation/progression of tumors 4. Receptor‐type protein tyrosine phosphatase κ (PTPRK), which belongs to a subfamily of membrane‐bound protein tyrosine phosphatases (PTPs), is expressed as a single precursor protein 5. Upon the single proteolytic cleavage catalyzed by furin protease 6, the resultant carboxyl‐terminal portion (P‐subunit) is anchored to cell membrane, recognizes the phosphorylated tyrosine residues of its target proteins, and catalyzes their dephosphorylation. The amino‐terminal portion (E‐subunit) acts as the extracellular subunit and includes a meprin/A5/m (MAM) domain, an immunoglobulin‐like domain and four fibronectin III‐like repeats; it associates with the P‐subunit non‐covalently. Like the other PTPs, PTPRK might attenuate the activation of various signaling pathways evoked by tyrosine phosphorylation of cellular proteins through the removal of their phosphate groups. For instance, PTPRK has been shown to dephosphorylate epidermal growth factor receptor (EGFR) and subsequently prohibits epidermal growth factor (EGF)‐mediated cell proliferation of human mammary epithelial cells and keratinocytes 7, 8, 9.

Notably, several lines of evidence indicate that PTPRK acts as a putative tumor suppressor. In support of this notion, it has been described that a loss of 6q22‐23 where the *PTPRK* locus is located is frequently detectable in patients with certain malignancies such as sporadic endocrine pancreatic tumors and juvenile intestinal carcinoma regardless of hereditary and inflammatory disease‐related factors 10, 11. Agarwal *et al*. 12 found a loss of function mutation within *PTPRK* in glioblastoma patients. Stevenson *et al*. 13 reported the presence of DNA methylation‐induced epigenetic silencing of *PTPRK* in certain blood malignancies. Consistent with these observations, a transposon‐mediated mutagenic screening revealed that mutation and/or dysregulation of *Ptprk* as well as *Apc*,* Pten*, and *Smad4* increases the susceptibility to intestinal lesions including intraepithelial neoplasia, adenoma, and adenocarcinoma 14. In addition, Sun *et al*. 15, 16 described that knockdown of *PTPRK* promotes proliferation and migration of human breast and prostate cancer cells.

The cancer stem cell (CSC) hypothesis has become increasingly accepted and might provide a clue to the understanding of the precise molecular basis underlying cancer initiation, progression, metastasis, and recurrence 17, 18, 19. Similar to normal tissue stem cells, CSC‐like cells with a higher tumorigenic potential are resistant to anti‐cancer drugs as well as irradiation 20, 21, and thus reliable molecular marker(s) for identifying CSCs might be a promising molecular target to develop a novel therapeutic strategy for cancers. CD133 (also known as prominin‐1/prominin‐like 1) is a unique pentaspan‐transmembrane glycoprotein initially identified in CD34‐positive hematopoietic stem cells 22, 23. Recently, CD133 has been recognized as one of the molecular markers of stem/progenitor cells in various tissues including kidney, neuron, and pancreas 24, 25, 26, 27. For example, Zhu *et al*. described that CD133‐positive cells exist at the base of the intestinal crypts and have a capability to differentiate into intestinal epithelial cells and goblet cells 24, 25, 26, 27. In addition to cellular differentiation, CD133‐positive tumor cells have displayed CSC‐like behaviors such as a higher tumorigenic as well as self‐renewal potential and chemo‐resistance, indicating that CD133 is one of the reliable CSC markers 28, 29, 30, 31. In good agreement with these functional properties, the expression level of CD133 has a strong prognostic impact on patients with various malignant tumors 32, 33, 34.

Since Boivin *et al*. 35 found tyrosine phosphorylation of CD133 by Src family kinases, its biological significance in tumorigenesis has been investigated. Recently, Wei *et al*. 36 found that the phosphorylation level of CD133 is positively associated with a progression of human brain tumor through the activation of the phosphoinositide 3‐kinase–AKT pathway. According to our previous study, forced expression of a phosphomimetic mutant of CD133 potentiated the aggressive phenotypes of colon cancer cells. In accordance with these results, a lower expression of *PTPRK*, which dephosphorylates CD133 *in vitro*, was significantly correlated to a short progression‐free survival of the patients with colon cancer expressing a higher level of *CD133* 37. In the present study, we sought to examine the functional significance of the PTPRK–CD133 axis in colon cancer progression and found for the first time that PTPRK‐mediated dephosphorylation of CD133 suppresses the progression of colon cancer cells and also improves their anti‐cancer drug sensitivity at least in part through down‐regulation of pro‐oncogenic CD133–AKT pathway.

## Materials and methods

### Cell culture

Human colon cancer‐derived HT‐29 and SW480 cells, and human embryonic kidney‐derived 293T cells were cultured in Dulbecco's modified Eagle's medium (DMEM; Sigma‐Aldrich, St Louis, MO, USA) supplemented with 10% heat‐inactivated FBS (Invitrogen, Carlsbad, CA, USA) and 50 μg·mL^−1^ of penicillin/streptomycin (Sigma‐Aldrich) in a humidified atmosphere with 5% CO_2_ at 37 °C. Their identities were verified by a short tandem repeat assay.

### Forced expression and knockdown by lentiviral vectors

Lentivirus‐mediated transduction was performed as described previously 37, 38. In brief, 293T cells were co‐transfected with combinations of lentivirus packaging plasmids (MISSION Lentiviral packaging mix; Sigma‐Aldrich) together with the transducing plasmid carrying cDNA for enhanced green fluorescent protein (eGFP) or CD133, or for the expression of short hairpin RNA (shRNA) against *PTPRK* (pLKO.1; Sigma‐Aldrich) using FuGENE HD transfection reagent (Promega, Madison, WI, USA) according to the manufacturer's instructions. Following the preparation of the cell‐free culture supernatants that contain virus vectors, the indicated colon cancer cells were cultured with the conditioned medium supplemented with 25% (v/v) of the virus‐containing culture supernatants for 24 h at 37 °C. These shRNA‐transfected cells were selected by puromycin (1 μg·mL^−1^; Sigma‐Aldrich).

### Semi‐quantitative RT‐PCR

Total RNA was extracted from cells using Isogen reagent (Nippon gene, Tokyo, Japan) and 5 µg of total RNA was reverse‐transcribed by Superscript III reverse transcriptase (Invitrogen) according to the manufacturers' protocols. The resultant cDNA was used for PCR. Oligonucleotide primer sets used in this study were as follows: *CD133*, 5′‐TTCCAGAAGCTCTGAGGCAG‐3′ (forward) and 5′‐AGAAATACCCCACCAGAGGC‐3′ (reverse); *PTPRK*, 5′‐TTGTTGAGACCAGCACAAGC‐3′ (forward) and 5′‐AAGCCTTGGTAGGTCCGATT‐3′ (reverse); *GAPDH*, 5′‐ACCACAGTCCATGCCATCAC‐3′ (forward) and 5′‐TCCACCACCCTGTTGCTGTA‐3′ (reverse). *GAPDH* was used as an internal control. PCR products were separated on 1% agarose gels and visualized by ethidium bromide staining.

### Western blot analysis

Cells were lysed in a lysis buffer containing 50 mm Tris/HCl (pH 7.5), 150 mm NaCl, 1% NP‐40, 1 mm EDTA and a protease inhibitors cocktail (Calbiochem, San Diego, CA, USA). Equal amounts of cell lysates were separated by 10% SDS/PAGE under reducing condition and electro‐transferred onto a poly(vinylidene difluoride) membrane (Merck Millipore, Billerica, MA, USA). The membrane was probed with the primary antibodies against CD133 (W6C3B1; Miltenyi Biotec, Bergisch Gladbach, Germany), PTPRK (HPA054822; Sigma‐Aldrich), phospho‐AKT at Ser‐473 (no. 4060; Cell Signaling Technology, Danvers, MA, USA), AKT (no. 9272; Cell Signaling Technology), phospho‐Bad at Ser‐136 (no. 4366; Cell Signaling Technology), Bad (no. 9239; Cell Signaling Technology), cleaved caspase‐3 (no. 9664; Cell Signaling Technology), caspase‐9 (no. 9502; Cell Signaling Technology), poly(ADP‐ribose) polymerase (PARP) (no. 9532; Cell Signaling Technology), eGFP (GTX26673; Gene Tex, Irvine, CA, USA) or with actin (A5060; Sigma‐Aldrich) followed by incubation with the appropriate horseradish peroxidase‐conjugated anti‐mouse IgG (no. 7074; Cell Signaling Technology) or with anti‐rabbit IgG antibody (no. 7076; Cell Signaling Technology). Immuno‐reactive signals were visualized with the Immunostar LD detection system (Wako, Osaka, Japan) and ImageQuant LAS4000 mini Imager (GE Healthcare Bioscience, Pittsburgh, PA, USA) according to the manufacturer's protocols.

### Immunoprecipitation and western blot analysis

Cells were treated with pervanadate [0.3% (w/w) H_2_O_2_ and 100 μm sodium orthovanadate in PBS] and then lysed in the lysis buffer as described above. Cell lysates were incubated with monoclonal anti‐CD133 antibody (AC133; Miltenyi Biotech) and protein G‐Sepharose beads at 4 °C for 2 h. The immunoprecipitates were analyzed by western blotting with monoclonal antibodies against CD133 (W6C3B1; Miltenyi Biotech), phospho‐tyrosine (no. 9411; Cell Signaling Technology) and PTPRK (HPA054822; Sigma‐Aldrich) as described above.

### Soft agar colony formation

Cells (5 × 10^2^) were suspended in 2 mL of top layer containing DMEM/10% FBS/0.33% agar and poured into 30‐mm cell culture dishes with 2 mL of basal layer including DMEM/10% FBS/0.5% agar. Fourteen days after incubation, the number of colonies larger than 100 μm in diameter was counted under the microscope.

### Tumor xenograft model

Cells (1 × 10^6^) were subcutaneously inoculated into BALB/c nude mice (CREA Japan, Shizuoka, Japan) as described previously 37. All of the animal experiments were performed in accordance with the guidelines of Chiba Cancer Center Research Institute.

### Sphere formation

Cells were seeded into 96‐well round bottom low‐attachment plates (Sumitomo Bakelite Co., Ltd, Tokyo, Japan) at a density of 10 cells per well and cultured for 9 days in sphere‐forming medium composed of DMEM/Ham's F‐12 (Wako) supplemented with 2% MACS Neurobrew‐21 (Miltenyi Biotec), 20 ng·mL^−1^ of EGF (R&D Systems, Minneapolis, MN, USA) and 20 ng·mL^−1^ of fibroblast growth factor‐2 (FGF‐2; R&D Systems) in a humidified atmosphere containing 5% CO_2_ at 37 °C. Images were taken under the phase‐contrast microscope and then measured diameters of spheres using imagej software 39. Sphere volume (*V*) was calculated by the formula: *V* = 4/3π × [long diameter (μm)] × [short diameter (μm)]^2^.

### WST assay

Cells were seeded into 96‐well plates at a density of 500 cells per well and allowed to attach overnight. Cells were then incubated at 37 °C in a humidified atmosphere with 5% CO_2_. Cell viability was examined by using Cell Counting Kit‐8 reagent (Dojindo Molecular Technologies, Rockville, MD, USA) according to the manufacturer's suggestions.

### Trypan blue‐dye exclusion assay

Cells were seeded into six‐well plates at a density of 1.5 × 10^5^ cells per well and exposed to oxaliplatin at a final concentration of 20 μm. Forty‐eight hours after treatment, floating and adherent cells were collected and washed in ice‐cold 1× PBS. After brief centrifugation, cells were resuspended in fresh medium, mixed with an equal volume of 0.4% trypan blue solution (Sigma‐Aldrich), and then analyzed by automatic cell counter (TC20; Bio‐Rad, Hercules, CA, USA).

### Statistical analysis

Results are presented as mean ± SD. Data were compared using unpaired *t*‐test, one‐way ANOVA, and repeated‐measures two‐way ANOVA. Analyses were performed using ekuseru‐tokei 2010 software (Social Survey Research Information Co., Ltd, Tokyo, Japan) and a *P*‐value < 0.05 was considered to be significant.

## Results

### Knockdown of *PTPRK* potentiates tumorigenic ability of CD133‐positive colon cancer HT‐29 cells

**Figure 1:**
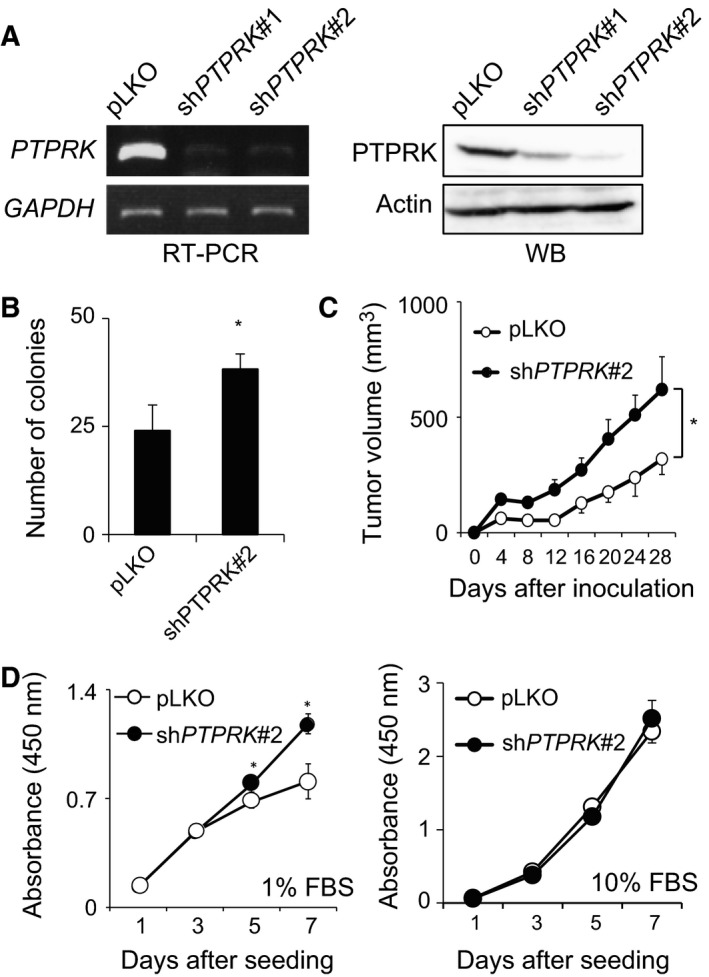
Knockdown of *PTPRK* potentiates the tumorigenic ability of CD133‐expressing colon cancer cells. (A) Establishment of *PTPRK*‐knocked down cells. Colon cancer HT‐29 cells were infected with the lentiviral vector harboring *PTPRK*‐targeting shRNAs (sh*PTPRK*#1 or sh*PTPRK*#2) or with the empty control (pLKO), and finally, puromycin‐resistant cells were obtained. Total RNA and cell lysates were prepared from the indicated cells (HT‐29/pLKO, HT‐29/sh*PTPRK*#1 and HT‐29/sh*PTPRK*#2) and analyzed for PTPRK by semi‐quantitative RT‐PCR (left panels) and western blotting (right panels), respectively. *GAPDH* or actin was used as an internal or a loading control, respectively. (B) Anchorage‐independent proliferation. pLKO and sh*PTPRK*#2 cells were cultured in soft agar medium (5 × 10^2^ cells/dish). Two weeks after incubation, a number of colonies (larger than 100 μm) were scored. Data show mean ± SD (*n *=* *3) and the asterisk indicates the statistical significance of difference (*P *<* *0.05, *t*‐test). (C) Xenograft tumor growth. The indicated cells (5 × 10^6^ cells) were subcutaneously inoculated into the flanks of BALB/c nude mice. The volumes of tumor‐derived pLKO (open circles) and sh*PTPRK*#2 (closed circles) cells were measured at the indicated time periods after inoculation. Data show mean ± SD (*n *=* *3) and the asterisk indicates the statistical significance of difference (*P *<* *0.05, *t*‐test). (D) HT‐29/pLKO (open circles) or HT‐29/sh*PTPRK*#2 (closed circles) were seeded into 96‐well plates (500 cells/well) and cultured with 1% FBS (left panel) or with 10% FBS (right panel) containing culture medium. Their proliferation rates were measured by WST assay at the indicated time points. Data show mean ± SD (*n* = 5) and asterisk indicates statistical significance of difference (*P *<* *0.05, repeated‐measures two‐way ANOVA).

To ask whether PTPRK could participate in the regulation of CD133‐mediated colon cancer progression, human colon cancer HT‐29 cells, which express endogenous CD133 (Fig. S1A), were infected with lentiviral vectors carrying shRNA against *PTPRK* (sh*PTPRK*#1 or sh*PTPRK*#2) or with the empty vector (pLKO). As shown in Fig. 1A, our shRNAs successfully reduced PTPRK expression at both mRNA and protein levels. Since sh*PTPRK*#2 down‐regulated PTPRK much more effectively than sh*PTPRK*#1, we have employed sh*PTPRK*#2 for further experiments.

Firstly, we examined the possible effect of *PTPRK* depletion on colony formation in soft agar medium. *PTPRK*‐depleted (HT‐29/sh*PTPRK*#2) or non‐depleted HT‐29 (HT‐29/pLKO) cells were maintained for 2 weeks in soft agar medium and then their colony numbers were scored after incubation. As seen in Fig. 1B, the number of colonies derived from HT‐29/sh*PTPRK*#2 cells was larger than that from HT‐29/pLKO cells. To confirm these results *in vivo*, HT‐29/pLKO and HT‐29/sh*PTPRK*#2 cells were inoculated into flanks of BALB/c nude mice. At the indicated time points after inoculation, their subcutaneous xenograft tumors were measured. As expected, the volume of tumors arising from HT‐29/sh*PTPRK*#2 cells was larger than that of HT‐29/pLKO cells (Fig. 1C), indicating that *PTPRK* gene silencing contributes to the tumor progression of colon cancer cells.

Since tumor growth promotion was observed in HT‐29/sh*PTPRK*#2 cells, their proliferation rate was determined by WST cell proliferation assay. As shown in Fig. 1D, the proliferation rate of HT‐29/sh*PTPRK*#2 cells was significantly faster than that of HT‐29/pLKO cells under a low serum concentration (1% of FBS), while knockdown of *PTPRK* had an undetectable effect on the proliferation rate of HT‐29 cells cultured under the standard serum concentration (10% of FBS).

### *PTPRK* gene silencing stimulates CD133‐mediated colon cancer growth

**Figure 2:**
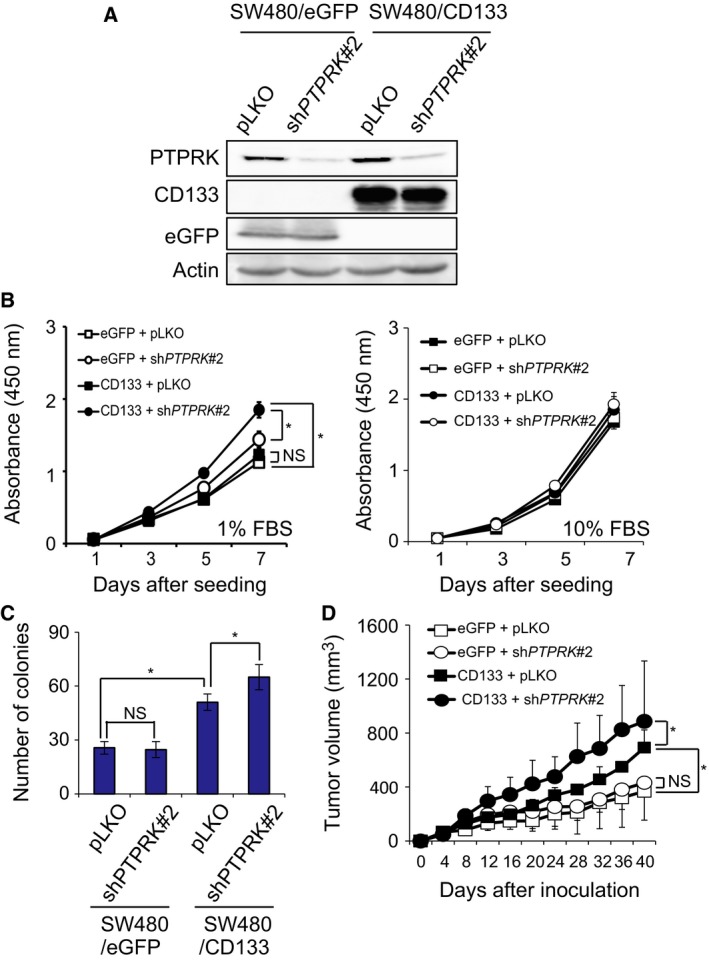
*PTPRK* gene silencing enhances pro‐oncogenic activity of CD133. (A) Forced expression of CD133 and silencing of *PTPRK*. CD133‐negative colon cancer SW480 cells were transduced with the lentiviral vector for CD133 or eGFP together with the sh*PTPRK*#2 or pLKO. Cell lysates were prepared from the indicated cells and analyzed for CD133, eGFP and PTPRK by western blotting. Actin was used as a loading control. (B) Aforementioned SW480 derivatives were seeded into 96‐well plates (500 cells per well) and cultured with 1% FBS (left panel) or with 10% FBS (right panel) containing culture medium. Their proliferation rates were examined by WST assay at the indicated time points. Data show mean ± SD (*n *=* *3) and the asterisk indicates the statistical significance of difference (*P *<* *0.05, repeated‐measure two‐way ANOVA). NS, not significant. (C) Anchorage‐independent proliferation. The indicated cells (500 cells per dish) were grown in soft agar medium for 2 weeks, and then the number of colonies (larger than 100 μm) was scored. Data show mean ± SD (*n *=* *3) and the asterisk indicates the statistical significance of difference (*P *<* *0.05, one‐way ANOVA). NS, not significant. (D) Xenograft tumor growth. The indicated cells (5 × 10^6^ cells) were subcutaneously inoculated into the flanks of BALB/c nude mice. Tumor volumes were measured at the indicated time periods after the inoculation. SW480/eGFP plus pLKO (open squares); SW480/eGFP plus sh*PTPRK* #2 (closed squares); SW480/CD133 plus pLKO (open circles); SW480/CD133 plus sh*PTPRK* #2 (closed circles). Data show mean ± SD (*n *=* *3) and the asterisk indicates the statistical significance of difference (*P *<* *0.05, one‐way ANOVA). NS, not significant.

To address whether PTPRK could attenuate the aggressive growth of colon cancer cells through the inhibition of CD133, we have employed CD133‐negative human colon cancer SW480 cells (Fig. S1B). SW480 cells were infected with the lentiviral vector for eGFP or CD133 to give SW480/eGFP or SW480/CD133 cells, respectively. These cells were subsequently infected with the lentiviral vector carrying sh*PTPRK*#2 or with pLKO. As shown in Fig. 2A, forced expression of eGFP and CD133, and forced depletion of *PTPRK* were confirmed by western blot analysis. We then determined the proliferation rates of these cells by WST cell proliferation assay at a low serum concentration (1% of FBS). Similar to HT‐29/sh*PTPRK*#2 cells, the proliferation rate of *PTPRK*‐depleted SW480/CD133 cells was significantly faster than that of non‐depleted SW480/CD133 cells (Fig. 2B, left panel). Additionally, *PTPRK* depletion had a negligible effect on the proliferation of SW480/eGFP cells under the lower serum condition (Fig. 2B, left panel). By contrast, knockdown of *PTPRK* had an undetectable effect on the proliferation rate of SW480/CD133 cells cultured with the standard 10% FBS‐containing medium (Fig. 2B, right panel).

We next determined the possible effect of *PTPRK* depletion on colony formation in soft agar medium. As shown in Fig. 2C, the number of colonies derived from SW480/CD133 cells was larger than that from SW480/eGFP control cells. Notably, knockdown of *PTPRK* in SW480/CD133 cells obviously increased number of colonies, whereas SW480/eGFP cells were unaffected by *PTPRK* depletion. Consistent with these results, SW480/CD133 cells developed larger xenograft tumors than SW480/eGFP cells, and silencing of *PTPRK* accelerated the growth of SW480/CD133‐derived tumors (Fig. 2D). In contrast to SW480/CD133‐derived tumors, SW480/eGFP‐derived tumors were unaffected by knockdown of *PTPRK*.

**Figure 3:**
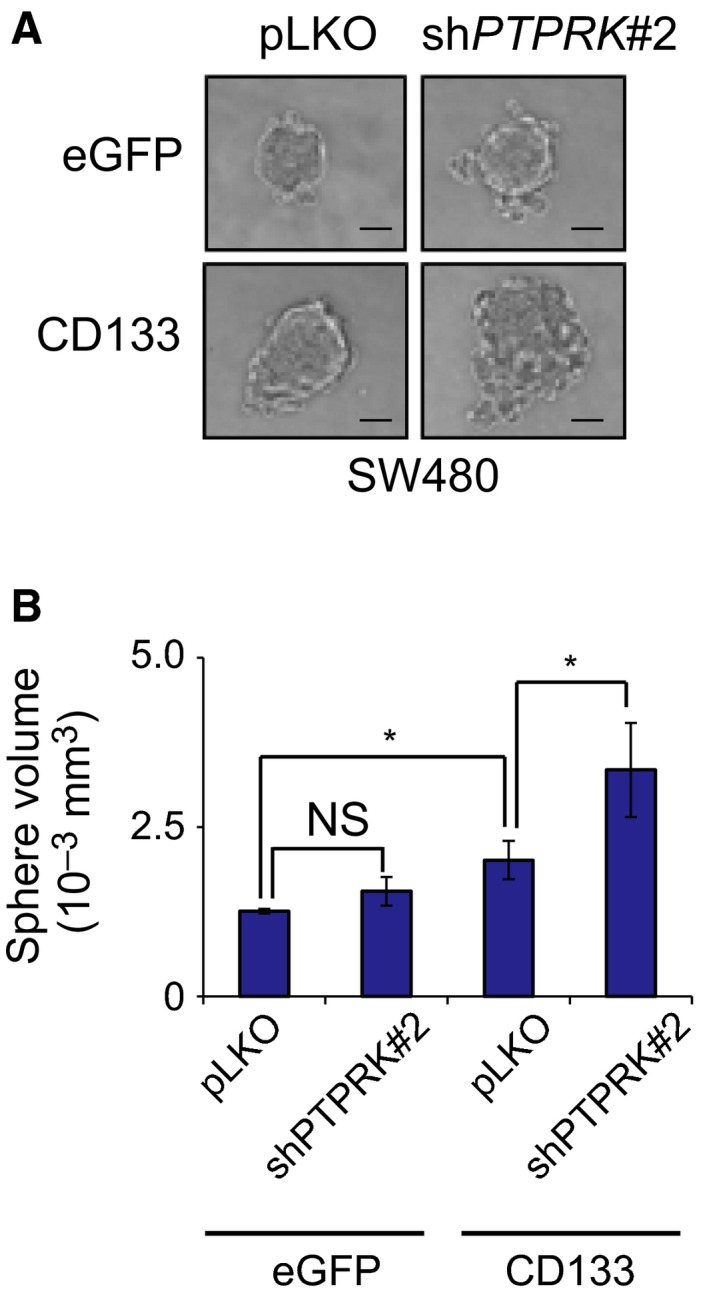
Knockdown of *PTPRK* enhances CD133‐dependent sphere‐forming ability of SW480 cells. (A,B) Sphere growth. SW480/eGFP plus pLKO, SW480/eGFP plus sh*PTPRK* #2, SW480/CD133 plus pLKO and SW480/CD133 plus sh*PTPRK* #2 (10 cells/well) were grown in serum‐free conditioned medium. Nine days after incubation, the representative images of spheres were taken (A), and their volumes were calculated (B). Bars indicate 50 μm (A). Data show mean ± SD (*n *=* *3) and the asterisk indicates the statistical significance of difference (*P *<* *0.05, one‐way ANOVA). NS, not significant.

We have further checked the possible effect of *PTPRK* knockdown on *in vitro* sphere formation. Sphere culture has been considered to be one of the surrogates of *in vitro* culture systems, which mimic the complicated microenvironments in tumor tissues *in vivo* 40. For this purpose, SW480/eGFP and SW480/CD133 cells were grown in the serum‐free sphere‐forming medium. Seven days after incubation, the volume of the spheres was measured. As shown in Fig. 3A,B, SW480/CD133 cells formed larger spheres relative to SW480/eGFP cells. Depletion of *PTPRK* had an undetectable effect on SW480/eGFP spheres, whereas *PTPRK*‐knocked down SW480/CD133 cells formed larger spheres as compared to non‐depleted SW480/CD133 cells. Similarly, *PTPRK*‐depleted HT‐29 spheres were larger than non‐depleted HT‐29 spheres (Fig. S2A,B).

Together, these observations suggest that PTPRK suppresses CD133‐mediated colon cancer growth both *in vitro* and *in vivo*.

### Silencing of *PTPRK* enhances CD133‐dependent phosphorylation of AKT and its downstream target Bad in SW480 cells

**Figure 4:**
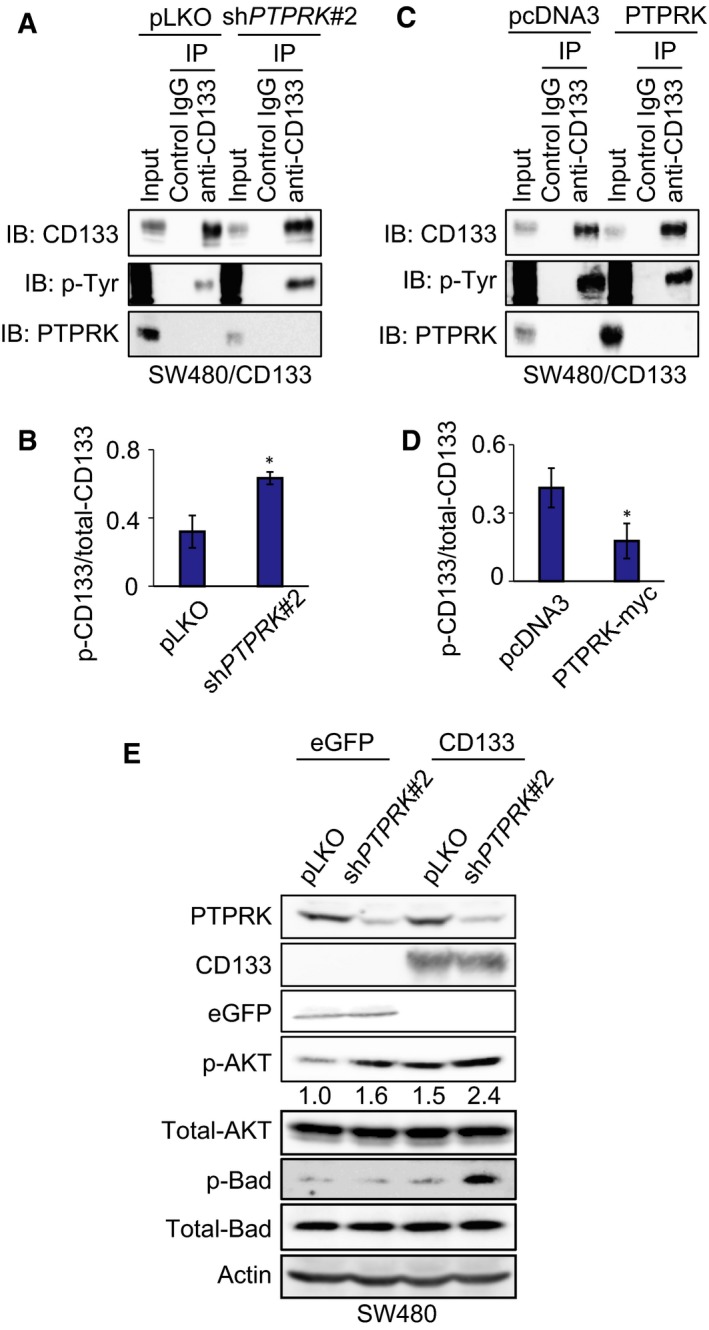
Depletion of *PTPRK* increases the level of phospho‐CD133 and leads to stimulated phosphorylation of AKT and a downstream target protein, Bad, in colorectal cancer cells. (A–D) Immunoprecipitation analysis. SW480/CD133 cells were infected with the lentiviral vector harboring *PTPRK*‐targeting shRNAs (sh*PTPRK*#2) or with mock control (pLKO). Cell lysates prepared from these SW480 derivatives were immunoprecipitated with anti‐CD133 antibody or with control IgG. Resultant immunoprecipitates were analyzed by western blotting with the indicated antibodies (A). The ratios of phospho‐CD133 (p‐CD133) to total CD133 from three independent experiments are indicated in (B). Alternatively, SW480/CD133 cells transfected with the expression plasmid for PTPRK or with pcDNA3 empty vector were processed for immunoprecipitation‐immunoblot analysis as mentioned above (C) and the ratios of p‐CD133/CD133 are shown in (D). Asterisks indicate statistical significance of difference (*P *<* *0.05, *t*‐test). (E) Western blot analysis. Cell lysates (30 μg) prepared from the indicated SW480 derivatives were analyzed by immunoblot with the indicated antibodies. Actin was used as a loading control. Relative band intensities of p‐AKT standardized to those of total‐AKT were also indicated.

Recently, we have described that tyrosine phosphorylation of CD133 (p‐CD133) contributes to aggressive tumor growth of colon cancer cells through the activation of the pro‐oncogenic AKT pathway 37. These findings prompted us to examine whether PTPRK‐mediated dephosphorylation of CD133 could attenuate the pro‐oncogenic AKT pathway in colon cancer cells. Towards this end, cell lysates prepared from non‐depleted and *PTPRK*‐depleted SW480/CD133 cells were immunoprecipitated with control IgG or with anti‐CD133 antibody followed by western blotting with anti‐phospho‐tyrosine antibody. As clearly seen in Fig. 4A,B, silencing of *PTPRK* significantly increased the p‐CD133 level. Consistent with these results, forced expression of PTPRK in SW480/CD133 cells resulted in a remarkable reduction of p‐CD133 level (Fig. 4C,D).

We next examined whether PTPRK could modulate AKT phosphorylation (p‐AKT) in CD133‐expressing SW480 cells. For this purpose, cell lysates were prepared from the indicated cells and analyzed for p‐AKT by western blotting. As shown in Fig. 4E, forced expression of CD133 in SW480 cells enhanced p‐ATK level at Ser‐473 as compared to that of SW480/eGFP cells. As expected, CD133‐induced phosphorylation of AKT at Ser‐473 was further stimulated in *PTPRK*‐depleted SW480/CD133 cells. The expression level of total AKT remained unchanged in response to the exogenous CD133 and *PTPRK* gene silencing.

To determine whether the pro‐oncogenic AKT signaling pathway could be activated under our experimental conditions, we checked the phosphorylation status of its downstream target Bad at Ser‐136 (p‐Bad). As shown in Fig. 4E, a remarkable up‐regulation of p‐Bad was detectable in *PTPRK*‐depleted SW480/CD133 cells (Fig. 4E). Forced expression of CD133 alone had an undetectable effect on p‐Bad. Similar results were also obtained in HT‐29/sh*PTPRK*#2 cells (Fig. S3). These observations suggest that PTPRK has the ability to impair the pro‐oncogenic CD133–AKT signaling pathway in colon cancer cells.

### Depletion of *PTPRK* confers resistance of CD133‐expressing SW480 cells to oxaliplatin

**Figure 5:**
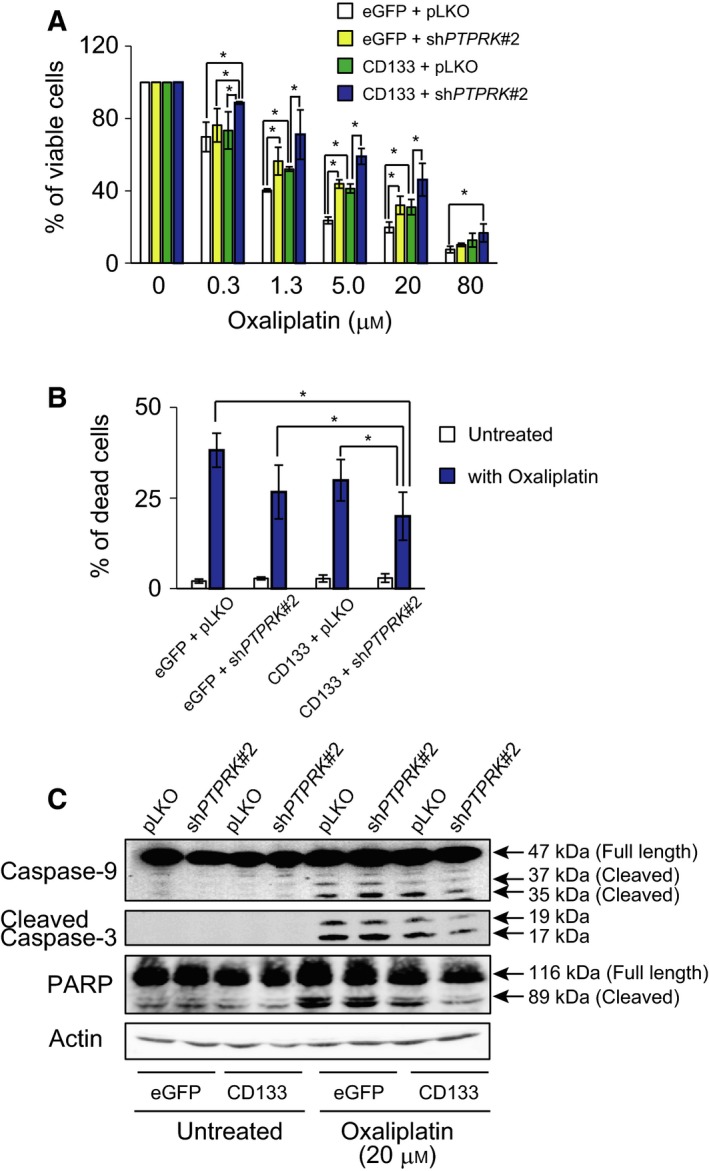
Silencing of *PTPRK* reduces oxaliplatin sensitivity of CD133‐expressing colon cancer cells. (A) WST cell survival assay. SW480 derivatives were treated with indicated concentrations of oxaliplatin for 48 h and their viability was measured by WST assay. Data represent mean ± SD (*n* = 5) and asterisks indicate statistical significance of difference (*P *<* *0.05, one‐way ANOVA). (B) Trypan blue dye‐exclusion assay. The indicated cells were exposed to 20 μm of oxaliplatin (blue bars) or left untreated (open bars). Forty‐eight hours after treatment, floating and attached cells were harvested and processed for trypan blue assay. Data represent mean ± SD (*n* = 3) and asterisks indicate statistical significance of difference (*P *<* *0.05, one‐way ANOVA). (C) Western blot analysis. The indicated cells were exposed to 20 μm of oxaliplatin or left untreated. Forty‐eight hours after treatment, cell lysates (30 μg) were prepared from floating and attached cells and analyzed by immunoblot with the indicated antibodies. Actin was used as a loading control.

Given that p‐Bad inhibits apoptotic cell death induced by the intrinsic mitochondrial pathway 41, we sought to investigate whether PTPRK could be implicated in CD133‐associated anti‐cancer drug resistance of colon cancer cells. For this purpose, non‐depleted and *PTPRK*‐depleted SW480/CD133 cells were treated with the indicated concentrations of oxaliplatin, which is commonly used in a standard chemotherapy for colon cancer patients. Forty‐eight hours after treatment, cell viability was assessed by WST assay. As shown in Fig. 5A, SW480/CD133 cells showed an enhanced viability as compared to SW480/eGFP cells following oxaliplatin exposure. Moreover, the viability of SW480/CD133 cells was further increased by *PTPRK* gene silencing in response to oxaliplatin. In support of these results, oxaliplatin‐induced cell death was obviously prohibited by knockdown of *PTPRK* as examined by trypan blue dye‐exclusion assay (Fig. 5B).

The trypan blue dye‐exclusion assay is an insensitive assay of cell death, particularly for early apoptosis, when compared with annexin V and propidium iodide staining method. To examine whether the oxaliplatin‐induced cell death would be mediated by apoptotic cell death, the indicated cells were treated with or without 20 μm of oxaliplatin, and then cell death‐related cleaved caspase‐9, caspase‐3 and PARP were examined by western blotting. As shown in Fig. 5C, oxaliplatin‐induced proteolytic cleavage of caspase‐9, caspase‐3 and PARP was detectable in both SW480/eGFP and SW480/CD133. Of note, *PTPRK* depletion in SW480/CD133 cells resulted in a massive decrease in the amounts of cleaved caspase‐9, caspase‐3 and PARP in response to oxaliplatin as compared to those of non‐depleted SW480/CD133 cells exposed to oxaliplatin.

Collectively, these results indicate that PTPRK‐mediated prohibition of the pro‐oncogenic CD133–AKT pathway improves oxaliplatin sensitivity of CD133‐expressing cancer cells.

## Discussion

**Figure 6:**
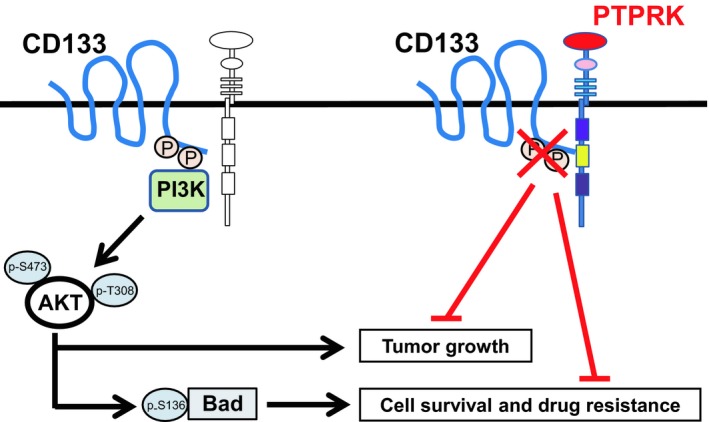
Summary of the present study.

In the present study, we have found for the first time that shRNA‐mediated knockdown of *PTPRK* significantly augments tumor growth and markedly reduces the anti‐cancer drug oxaliplatin sensitivity of CD133‐expressing colon cancer cells. Our expression studies revealed that *PTPRK* silencing enhances the phosphorylation of CD133, AKT and its downstream target Bad. Thus, this is suggestive that forced depletion of *PTPRK* contributes to the aggressive properties of CD133‐positive colon cancer cells through the potentiation of the pro‐oncogenic CD133–AKT pathway (Fig. 6).

According to our present results, depletion of *PTPRK* conferred resistance to oxaliplatin in SW480/CD133 cells in association with the marked accumulation of p‐CD133. Our previous study demonstrated that phosphor‐mimetic CD133 mutant protein leads to an increase in phosphorylation of AKT and subsequently activates its enzymatic activity 37. In support of this observation, *PTPRK*‐silenced SW480/CD133 cells showed the highest level of p‐Bad at Ser‐136, which is phosphorylated by AKT 42. Bad can be also phosphorylated at Ser‐112 mediated by the Ras–mitogen‐activated protein kinase pathway 43, and the phosphorylation of both serine residues inhibits the pro‐apoptotic function of Bad via the interaction with 14‐3‐3 proteins but not with BCL‐X_L_ 44. Our present result, however, demonstrated that neither knockdown of *PTPRK* nor forced expression of CD133 affects the level of p‐Bad at Ser‐112 in SW480 cells (Fig. S4). Consistent with these observations, Ma *et al*. 45 described that the activation of the pro‐survival AKT–Bad pathway but not of the mitogen‐activated protein kinase pathway is tightly linked to the drug‐resistant phenotype of the CD133‐positive subset in hepatocellular carcinoma HuH7 cells. We have therefore proposed a model in which a PTPRK–CD133 axis exists and prevents cell death induced by certain anti‐tumor drugs through activation of the AKT–Bad pro‐survival pathway. Furthermore, we have demonstrated that knockdown of *PTPRK* enhances proliferation of SW480/CD133 cells but not of SW480/eGFP cells under low serum conditions. Edinger *et al*. 46 described that mammalian target of rapamycin, another downstream target of AKT, plays a pivotal role in cell survival through increased nutrient uptake. These studies collectively suggest that the PTPRK–CD133 axis is also implicated in cell survival and an adaptation of cancer cells to poor microenvironment in tumor tissue *in vivo*; however, further examination of metabolic pathway activation in *PTPRK*‐depleted CD133‐positive cancer cells under lower nutrient and/or hypoxic conditions is required.

The present study showed that knockdown of *PTPRK* accelerated xenografted tumor growth of SW480/CD133 cells but not of CD133‐negative SW480/eGFP cells accompanied by a larger amount of p‐AKT. Bläker *et al*. 10 described that *PTPRK* is recurrently mutated in precancerous lesions of certain intestinal carcinoma patients. Zhu *et al*. 47 demonstrated that CD133‐positive stem/progenitor cells possess the traits of CSCs in response to oncogenic insults. Although natural ligands for CD133, to our knowledge, have not been identified, our present results are suggestive that the abnormality of *PTPRK* caused by loss‐of‐function mutation and/or deletion is one of the critical triggers for CD133‐positive stem/progenitor cells to transform cancerous cells *in vivo*. According to our previous study 37, EGF could stimulate phosphorylation of CD133 in colon cancer cells. The pro‐oncogenic EGF/EGFR signaling pathway, which activates AKT phosphorylation 48, is suppressed by PTPRK through the promotion of EGFR dephosphorylation 8, 49. Intriguingly, a clear induction of p‐AKT was observed in *PTPRK*‐silenced SW480/eGFP cells, but it is insufficient to further augment *in vivo* tumor growth of CD133‐negative SW480/eGFP cells. Additionally, a sharp increase in p‐AKT was observed in *PTPRK*‐silenced SW480/CD133 cells. We therefore hypothesized that the PTPRK–CD133 axis is implicated in the pro‐oncogenic EGF/EGFR signaling pathway. Detailed analysis of EGF/EGFR signal status in *PTPRK*‐silenced colon cancer cells might help to better understand mechanisms of CD133 stimulation by EGF.

Another important finding of our present study was that *PTPRK*‐depleted SW480/CD133 cells form tumorspheres much more efficiently than non‐depleted SW480/CD133 cells. Unexpectedly, PTPRK has been shown to be highly expressed in breast and prostate cancer tissues as compared to their corresponding non‐tumor ones 15, 16, raising the possibility that the tumor suppressive function(s) but not the expression of PTPRK might be impaired in cancer cell‐derived spheres as well as cancer tissues. Previously, it has been shown that the active site within the phosphatase domain of PTPs is highly susceptible to oxidation caused by reactive oxygen species (ROS), and the oxidized PTPs are no longer able to catalyze dephosphorylation 9, 50. It has been well documented that various types of cancer cells produce a large amount of ROS, which is implicated in the growth promotion of cancers 51. For instance, Le Belle *et al*. 52 demonstrated that a higher level of ROS stimulates the proliferation and self‐renewal of neuronal stem cells. Qu *et al*. 53 found that prostate cancer cell spheres generate a large amount of ROS relative to their parental adherent monolayer cultures, which augments their growth through ROS‐mediated activation of the interleukin 6–signal transducer and activator of transcription 3 pathway. Based on our current results, the intracellular ROS level was significantly increased during sphere formation of SW480 cells (Fig. S5), suggesting that ROS inhibits PTPRK and thereby facilitates CD133‐mediated sphere formation. We also speculate that the reactivation of PTPRK might suppress the sphere forming ability of SW480/CD133 cells. Further studies of ROS scavengers, such as *N*‐acetyl cysteine and melatonin, will be required to adequately address this issue.

In conclusion, our present results strongly suggest that PTPRK‐mediated dephosphorylation of CD133 plays an essential role in the progression and drug resistance of colon cancer at least in part through down‐regulation of the pro‐oncogenic AKT–Bad pathway, and this regulatory mechanism might provide a clue to develop a novel and promising strategy for the better treatment of colon cancer patients.

## Conflict of interest

The authors declare no conflict of interest.

## Author contributions

OS conceived and supervised the study; MM and OS designed experiments; MM, YM, KU, TOg, MN and HY performed experiments; OS, TOz, HS, NT and YN analyzed and interpreted data; MM, OS and TOz wrote the manuscript.

## Supporting information


**Fig. S1.** Expression levels of PTPRK and CD133 in human colon cancer cells.

**Fig. S2.** Knockdown of *PTPRK* accelerates sphere growth of HT‐29 cells.

**Fig. S3.** Knockdown of *PTPRK* stimulates phosphorylation of AKT and its target proteins in HT‐29 cells.

**Fig. S4.** Knockdown of PTPRK and forced‐expression of CD133 has a negligible effect on phosphorylation of Bad at Ser‐112 in SW480 cells.

**Fig. S5.** A higher production of reactive oxygen species (ROS) in colon cancer‐derived spheres.
