# Supplementary material for: PTPRK suppresses progression and chemo‐resistance of colon cancer cells via direct inhibition of pro‐oncogenic CD133
Source: FEBS Open Bio. 2019 Apr 18;9(5):935–46. doi: 10.1002/2211-5463.12636 (PMC6487712; doi:10.1002/2211-5463.12636)
Supplement: Supplementary file 1 — Fig. S1. Expression levels of PTPRK and CD133 in human colon cancer cells. [file FEB4-9-935-s001.pdf]

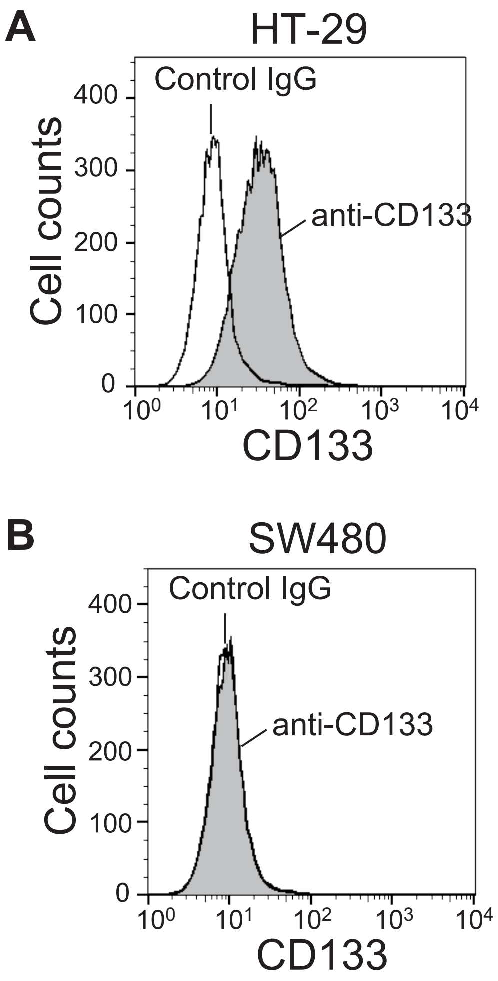


**Supplementary Figure S1. Expression levels of PTPRK and CD133 in human colon cancer cells.** Flow cytometric analysis. HT-29 (A) and SW480 cells (B) were treated with PE-labeled anti-CD133 monoclonal antibodies (293C3, purchased from Miltenyi Biotech, Bergisch Gladbach, Germany) and then expression level of CD133 was analyzed by FACS Calibur cytometer (BD Bioscience, Franklin Lakes, NJ, USA) and FlowJo software (Tree Star, Ashland, OR, USA).
