# Supplementary material for: PTPRK suppresses progression and chemo‐resistance of colon cancer cells via direct inhibition of pro‐oncogenic CD133
Source: FEBS Open Bio. 2019 Apr 18;9(5):935–46. doi: 10.1002/2211-5463.12636 (PMC6487712; doi:10.1002/2211-5463.12636)
Supplement: Supplementary file 2 — Fig. S2. Knockdown of PTPRK accelerates sphere growth of HT‐29 cells. [file FEB4-9-935-s002.pdf]

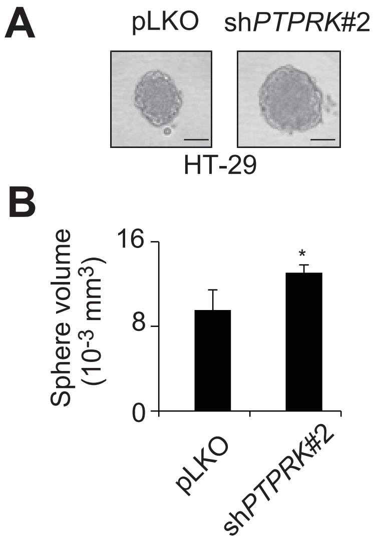

Supplementary Figure S2. Knockdown of PTPRK accelerates sphere growth of HT-29 cells. Sphere growth. pLKO- or shPTPRK#2-transduced HT-29 cells were seeded into 96-well round bottom plates (10 cells in each well) and cultured in serum-free conditioned-medium. Nine days after incubation, images of spheres were taken (A) and their volumes were calculated by orthogonal diameters (B). Bars indicate 100 μm (A). Data shows mean ± SD (n = 5) and asterisk indicates statistical significance of difference ($P < 0.05$, *t*-test).
