# Supplementary material for: PTPRK suppresses progression and chemo‐resistance of colon cancer cells via direct inhibition of pro‐oncogenic CD133
Source: FEBS Open Bio. 2019 Apr 18;9(5):935–46. doi: 10.1002/2211-5463.12636 (PMC6487712; doi:10.1002/2211-5463.12636)
Supplement: Supplementary file 3 — Fig. S3. Knockdown of PTPRK stimulates phosphorylation of AKT and its target proteins in HT‐29 cells. [file FEB4-9-935-s003.pdf]

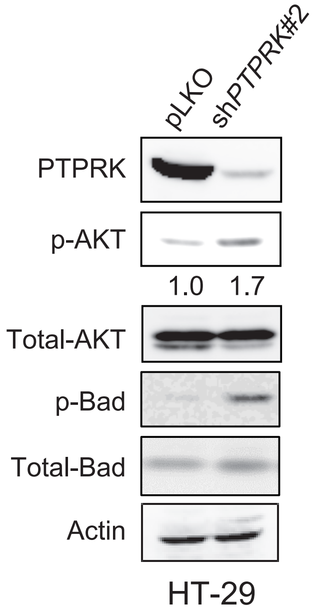

Supplementary Figure S3. Knockdown of PTPRK stimulates phosphorylation of AKT and its target proteins in HT-29 cells.

Immunoplot analysis. Cell lysates (30 μg) prepared from the indicated HT-29 derivatives were analyzed by immunoblot with the indicated antibodies. Actin was used as a loading control. Relative band intensities of phosphorylated-AKT (p-AKT) standardized by those of total-AKT were also indicated.
