# Supplementary material for: PTPRK suppresses progression and chemo‐resistance of colon cancer cells via direct inhibition of pro‐oncogenic CD133
Source: FEBS Open Bio. 2019 Apr 18;9(5):935–46. doi: 10.1002/2211-5463.12636 (PMC6487712; doi:10.1002/2211-5463.12636)
Supplement: Supplementary file 4 — Fig. S4. Knockdown of PTPRK and forced‐expression of CD133 has a negligible effect on phosphorylation of Bad at Ser‐112 in SW480 cells. [file FEB4-9-935-s004.pdf]

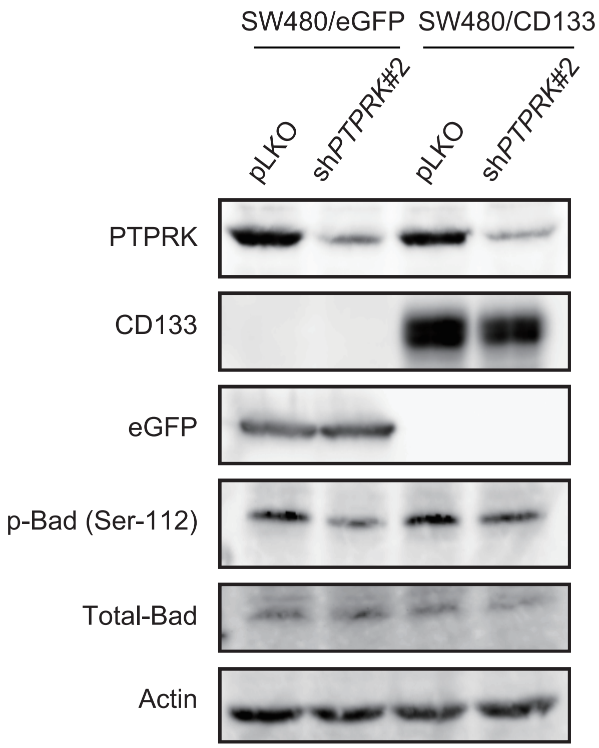

Supplementary Figure S4. Knockdown of PTPRK and forced-expression of CD133 has a negligible effect on phosphorylation of Bad at Ser-112 in SW480 cells.

Immunoplot analysis. Cells were lysed in a lysis buffer containing 50 mM Tris-HCl (pH 7.5), 150 mM NaCl, 1% NP-40, 1 mM EDTA and a protease inhibitors cocktail (Calbiochem, San Diego, CA, USA). Cell lysates (30 μg/lane) were separated by SDS-PAGE under reduced condition and electro-transferred onto a PVDF membrane (Merck Millipore, Billerica, MA, USA). The membrane was probed with the primary antibodies against CD133 (W6C3B1, Miltenyi biotech, Bergisch Gladbach, Germany), PTPRK (HPA054822, Sigma-Aldrich, St. Louis, MO, USA), phospho-Bad at Ser-112 (ab129192, Abcam, Tokyo, Japan), Bad (#9239, Cell Signaling Technology), eGFP (GTX26673, Gene Tex, Irvine, CA, USA) or with Actin (A5060, Sigma-Aldrich) followed by the incubation with the appropriate horseradish peroxidase-conjugated anti-mouse IgG (#7074, Cell Signaling Technology) or with anti-rabbit IgG antibody (#7076, Cell Signaling Technology). Immuno-reactive signals were visualized by Immunostar LD detection system (Wako, Osaka, Japan) and ImageQuant LAS4000 mini Imager (GE Healthcare Bioscience, Pittsburgh, PA, USA) according to the manufacturer' s protocols.
