# Supplementary material for: PTPRK suppresses progression and chemo‐resistance of colon cancer cells via direct inhibition of pro‐oncogenic CD133
Source: FEBS Open Bio. 2019 Apr 18;9(5):935–46. doi: 10.1002/2211-5463.12636 (PMC6487712; doi:10.1002/2211-5463.12636)
Supplement: Supplementary file 5 — Fig. S5. A higher production of reactive oxygen species (ROS) in colon cancer‐derived spheres. [file FEB4-9-935-s005.pdf]

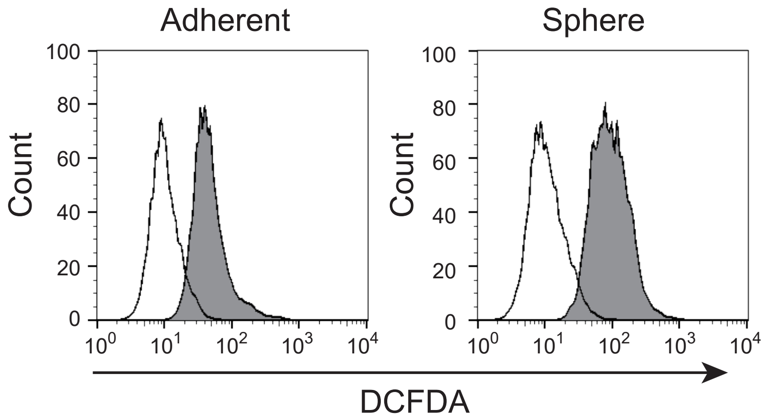

Supplementary Figure S5. A higher production of reactive oxygen species (ROS) in colon cancer-derived spheres.

SW480 cells were cultured in the conventional serum-containing medium (Adherent) or in serum-free conditioned medium (Sphere) for three days as described in Materials and Methods. These cells were harvested and stained with an itracellular ROS indicator (DCFDA) according to the manufacturer’ s instruction. The fluorescence of cells treated with DCFDA (grey) or without DCFDA (white) was observed by FACS Calibur cytometer (BD Bioscience, Franklin Lakes, NJ, USA) and FlowJo software (Tree Star, Ashland, OR, USA). The representative histogram is shown.
